# Chemo-Enzymatic Synthesis of Silybin and 2,3-Dehydrosilybin Dimers

**DOI:** 10.3390/molecules19044115

**Published:** 2014-04-02

**Authors:** Eva Vavříková, Jan Vacek, Kateřina Valentová, Petr Marhol, Jitka Ulrichová, Marek Kuzma, Vladimír Křen

**Affiliations:** 1 Institute of Microbiology, Academy of Sciences of the Czech Republic, Vídeňská 1083, CZ-142 20 Prague 4, Czech Republic; E-Mails: vavrikova@biomed.cas.cz (E.V.); kata.valentova@email.cz (K.V.); rtepm@seznam.cz (P.M.); kuzma@biomed.cas.cz (M.K.); 2 Department of Medical Chemistry and Biochemistry, Faculty of Medicine and Dentistry, Palacký University, Hněvotínská 3, CZ-775 15 Olomouc, Czech Republic; E-Mails: jan.vacek@upol.cz (J.V.); jitka.ulrichova@upol.cz (J.U.)

**Keywords:** flavonolignan, silybin, silibinin, dimerization, electrochemistry, antioxidant, microsomal lipid peroxidation, HUVEC, cytotoxicity

## Abstract

Divalent or multivalent molecules often show enhanced biological activity relative to the simple monomeric units. Here we present enzymatically and chemically prepared dimers of the flavonolignans silybin and 2,3-dehydrosilybin. Their electrochemical behavior was studied by *in situ* and *ex situ* square wave voltammetry. The oxidation of monomers and dimers was similar, but adsorption onto the electrode and cell surfaces was different. A 1,1-diphenyl-2-picrylhydrazyl (DPPH) and an inhibition of microsomal lipoperoxidation assay were performed with same trend of results for silybin and 2,3-dehydrosilybin dimers. Silybin dimer showed better activity than the monomer, while on the contrary 2,3-dehydrosilybin dimer presented weaker antioxidant/antilipoperoxidant activity than its monomer. Cytotoxicity was evaluated on human umbilical vein endothelial cells, normal human adult keratinocytes, mouse fibroblasts (BALB/c 3T3) and human liver hepatocellular carcinoma cell line (HepG2). Silybin dimer was more cytotoxic than the parent compound and in the case of 2,3-dehydrosilybin its dimer showed weaker cytotoxicity than the monomer.

## 1. Introduction

**Figure 1:**
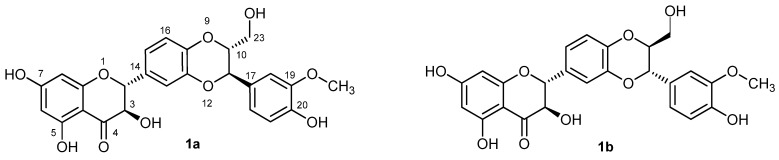
Silybin A (**1a**) and silybin B (**1b**).

The flavonolignan silybin (**1**), an antioxidant and hepatoprotectant, is a major bioactive component of the extract from the milk thistle [*Silybum marianum* (L.) Gaertn. (Asteraceae)] denoted as silymarin [1]. Silybin occurs in silymarin as an approximately equimolar mixture of two diastereoisomers: silybin A (**1a**) and silybin B (**1b**) (Figure 1) [2]. 2,3-Dehydrosilybin (**6**) occurs in silymarin in minor amounts (also as a mixture of enantiomers) presumably resulting from spontaneous oxidation of silybin [3], and it has significantly higher anticancer [4,5] and antioxidant [6,7] activity than silybin.

Silybin and its congeners, being effective chemoprotectants, have been used for a diverse range of semisynthetic modifications, both chemical and enzymatic. Enzymatic methods are more suitable than chemical ones due to the sensitivity of these flavonoids to oxidation and extreme pH.

Acylation at position C-23, which does not participate in the antioxidant activity of silybin [7,8], has been accomplished both with chemical [9] and enzymatic [10,11] methods and produced new antiviral and antitumor compounds. Enzymatic acylation/deacylation at the C-23 OH also enabled the first diastereomeric separation at the preparatory scale [12]. On the other hand, modification (e.g., methylation) of C-7 OH, bearing a pro-oxidant potential, significantly improves the antiradical activity of silybin [8].

Silybin has already been covalently conjugated with other drugs, leading to hybrid or co-drug molecules. This was recently exemplified by the preparation of a tacrine-silybin co-drug aiming to lower the hepatotoxicity of tacrine while maintaining its acetylcholinesterase inhibitory effects that are used in the treatment of Alzheimer’s disease [13]. Silybin was also linked at C-23 by a phosphodiesteric moiety to various molecules aiming to improve its biological properties [14].

Silybin dimerization has also been achieved: the first oxidative dimerization of partially protected silybin with laccase yielded C-C and C-O dimers [15]. This study was focused on describing the mechanism of oxidative attack on silybin. Later, Theodosiou *et al.* [11] detected (only by MS) silybin diester dimers linked with dicarboxylic acids to the 23-OH as byproducts of the synthesis of silybin acylated derivatives using lipase-catalyzed esterification. Both of the above studies were performed with natural silybin (**1**), *i.e.*, an equimolar mixture of silybin A (**1a**) and B (**1b**). Using this mixture would not cause a problem in the antioxidant studies, as we have demonstrated that both **1a** and **1b** behave in a very similar way towards radical and/or oxidative attack in an isotropic milieu [16]. Nevertheless, interaction with biological systems involves interactions with the 3D structures and here the stereochemistry of the ligands is of the utmost importance. Dimerization of the diastereomeric silybin mixture could lead in theory to three compounds (AA, BB, AB), however, their proportions are rather unpredictable as the lipases typically display stereoselectivity [12] towards **1a** and **1b**, and the MS analysis used [11] could not deconvolute the mixture. A typical example of the differing biological activity of **1a** and **1b** is the fact that silybin B (**1b**) interacts with estrogen receptors, whereas silybin A (**1a**) is devoid of this activity [17].

Divalent or multivalent molecules often exhibit enhanced biological activity relative to the simple monovalent units [18]. It is not only the multivalency effect that modifies their biological activity (multi-ligand binding) but also their pharmacokinetic parameters, as shown recently in artemisinin dimers used as antimalarial drugs [19]. Sometimes an entirely new biological entity is created by dimerization, e.g., the oligomerization of ergot alkaloids lead to an entirely new antiplasmodial activity [20]. The dimerization of flavonoids, aiming at the preparation of more effective P-glycoprotein inhibitors, has been accomplished by the synthesis of apigenin homodimers linked with polyethylene glycol spacers [21]. This study also demonstrated that the length of the spacer is a critical parameter for the binding of dimers to the target protein(s).

We have decided to prepare defined dimers of silybin and 2,3-dehydrosilybin, including heterodimers using both the diester enzyme-catalyzed concept [22] and the diether (chemical) approach. The basic properties of these new compounds were determined by using antioxidant, electrochemical and toxicological assays to enable their further application in more complex biological studies.

## 2. Results and Discussion

### 2.1. Synthesis of Dimers

Lipase-mediated synthesis is a preferable and very effective method for the selective acylation of primary hydroxyl groups in non-aqueous media [23]. We used the divinyl esters of dicarboxylic acids as donors due to the fact that lipase-catalyzed reaction is faster and the reaction equilibrium is more shifted in favor of transesterification than in the reactions with free acids. Here we used Novozym 435—a lipase B from *Candida antarctica* immobilized on an acrylic resin, taking advantage of its specific selectivity and straightforward handling. Reactions were performed in dry acetonitrile, in which silybin was completely soluble (ca 12 g/L). In the case of enzymatic reactions silybin is typically not completely dissolved at the start of the reaction but it dissolves as the reactions progress. 

**Scheme 1:**
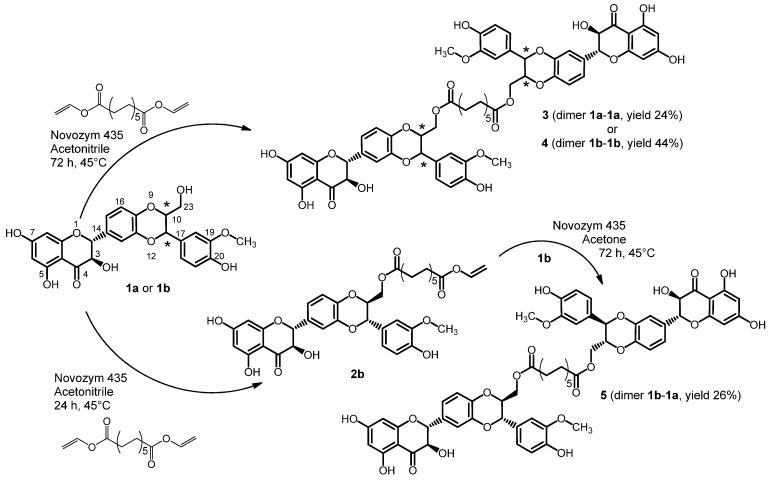
Lipase-catalyzed synthesis of optically pure silybin dimers.

The lipase-catalyzed acylation of the primary group of silybin A (**1a**) and B (**1b**) was used for preparation of the C-23 derivatives of symmetric dimers of silybin **3** and **4** (Scheme 1).

The preparation of the C-23 derivatives of silybin symmetric dimers **3** and **4** involves a lipase-catalyzed acylation of the primary hydroxyl group of silybin (Scheme 1). The ratio of the stoichiometric coefficients of the reactants silybin and divinylester of dodecandioic acid was 2.7:1. Dodecanedioic acid was converted to its divinyl ester in the presence of vinyl acetate and a catalytic amount of mercury(II) acetate according to [24]. The synthesis of asymmetric dimer **5** was comprised of two steps (Scheme 1). First, the synthesis of the activated acyl donor (12-vinyl dodecandioate-23-*O*-silybin B, **2b**) was accomplished by lipase-mediated esterification of the C-23 of **1a**.

The length of the aliphatic linker between the two units of silybin was optimized. Divinyl esters of succinic (C4), glutaric (C5) and adipic acid (C6) were reacted with silybin under Novozym 435 catalysis but no silybin dimers were formed. The short aliphatic chain did not allow the linkage of two units of silybin, even though monovinyl esters or hydrolyzed esters of silybin were formed. Hence, a divinyl ester of dodecanedioic acid (C12) was compatible with the stereochemistry of sterically demanding molecule of the silybin dimer and the yield of the prepared compounds differs from 24% to 44%. In the study of Theodosiou *et al.* [11], silybin dimers with aliphatic chains (C6, C12, C16) were synthesized and detected by HPLC/MS analysis as byproducts during the preparation of silybin acyl esters, however, the yields were very low (2.9%–6.2%).

**Scheme 2:**
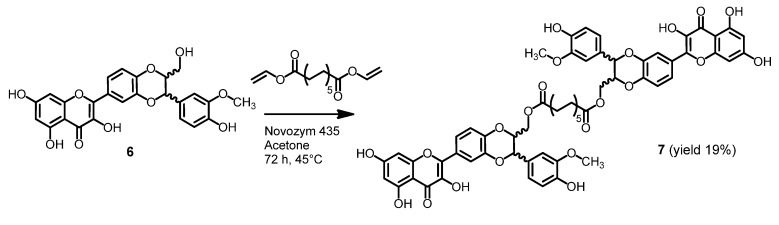
Lipase-catalyzed synthesis of 2,3-dehydrosilybin dimer.

The dimer of 2,3-dehydrosilybin (**7**) was also prepared in the presence of Novozym 435 with a 4:1 ratio of the stoichiometric coefficients of the reactants silybin and divinylester of dodecanedioic acid (Scheme 2). Due to paucity of enantiomerically pure dehydrosilybin the compound **7** was prepared from the enantiomeric mixture.

**Scheme 3:**
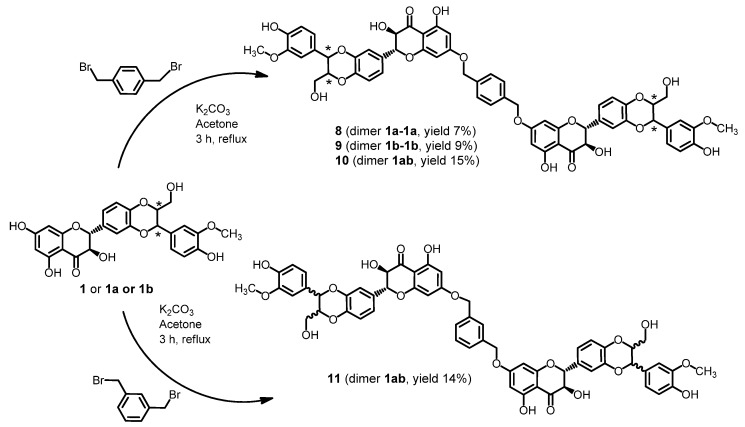
Synthesis of silybin dimers with ether spacer.

Chemical synthesis allowed us to prepare entirely new dimers due to the significantly higher reactivity of the hydroxyl group in the position C-7 of silybin in comparison to other hydroxyl groups. Reaction with *p*- or *m*-xylylene dibromide in the presence of K_2_CO_3_ was used for formation of silybin dimers **8**–**11** with etheric bridges (Scheme 3). In contrast to the flexible aliphatic linker of dimers **3**, **4**, **5**, **7** a xylyl moiety has a rigid structure.

All prepared dimers and monomers **1a**, **1b**, **6** were tested for electrochemical behavior using SWV and antioxidant activity using DPPH scavenging. Data (not shown) for both types of silybin dimers were comparable with that of the silybin monomers, hence, for following results and discussion silybin A (**1a**) and the product **3** were chosen (Table 1).

### 2.2. Stability of Dimers

The stability of the dimers in PBS and phosphate buffers (0.2 M, pH 7.4) was determined by HPLC after 24 h. In all cases, no decomposition of the ester or ether bonds was observed (data not shown). However, during longer incubation signs of instability (decomposition) were observed in compounds **4** and **5** and, therefore, these compounds were not further tested in biological assays.

### 2.3. Electrochemistry

**Figure 2:**
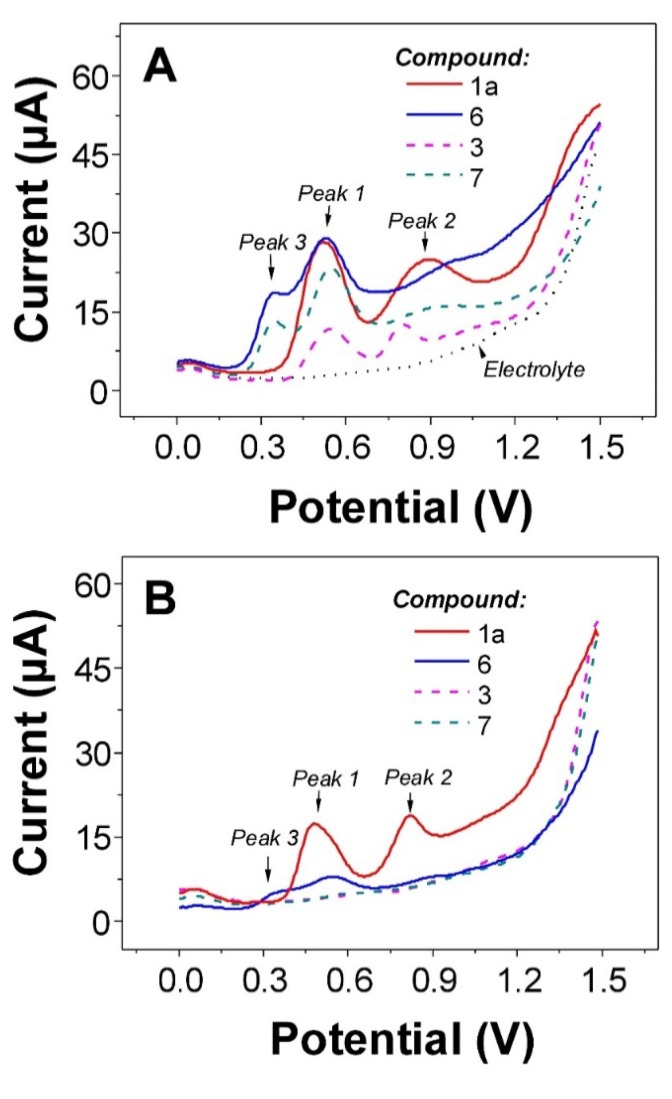
*In situ* (**A**) and *ex situ* (**B**) SW voltammograms of flavonolignans **1a**, **3, 6**, and **7**. Non-adsorbing species in panel (**B**), *i.e.*, flavonolignans **3** and **7**, providing the background very similar to “Electrolyte” in panel (**A**).

The electrochemical behavior of monomers **1a**, **6** and their dimers was studied using SWV under *in situ* and *ex situ* (adsorptive transfer) conditions according to the previously published protocol [7]. For **1a** and its dimers we can observed two well-developed oxidation peaks at *E*_p1_ ~ +0.55 V and *E*_p2_ ~ +0.87 V (Figure 2).

**Table 1:** Oxidation potentials (*E*_p_) and DPPH scavenging for monomeric flavonolignans (**1a**, **6**) and their dimers at pH 7.4.

Compound	Oxidation potential [V] * *vs.* Ag/AgCl/3 M KCl *n* = 3			DPPH [% of inhibition] ** *n* = 4
	*E*p_3_	*E*p_1_	*E*p_2_	33 µM
**1a**		0.55	0.90	6.6 ± 1.7 ^a^
**3**		0.56	0.83	7.1 ± 2.8 ^a^
**4**		0.57	0.83	9.0 ± 3.0 ^a^
**5**		0.55	0.83	7.0 ± 2.9 ^a^
**6**	0.37	0.54	0.92	82.9 ± 0.2 ^b^
**7**	0.36	0.57	0.99	33.4 ± 3.1 ^c^
**8**		0.56	0.81	8.5 ± 3.7 ^a^
**9**		0.57	0.83	8.9 ± 4.0 ^a^
**10**		0.56	0.80	8.1 ± 3.4 ^a^
**11**		0.52	0.87	10.7 ± 3.1 ^a^

* The values of oxidation potentials *E*p were estimated by SWV. The same values can be observed by CV with a difference of ±0.05 V. ** DPPH scavenging is expressed as absorbance change caused by 33 µM test compound—the higher the value, the better the scavenger. Values marked with the same letter are not significantly different (*p* < 0.05). *E*_p1_ ~ +0.55 V can be ascribed to oxidation of C-20 OH; *E*_p2_ ~ +0.87 V relates to oxidation of resorcinol moiety (ring A), probably C-5 OH; *E*_p3_ ~ +0.37 V can be ascribed to oxidation of C-3 OH (**6**, **7**).

The first peak can be ascribed to oxidation of C-20 hydroxyl group at ring E. Second oxidation step is connected to the oxidation of resorcinol moiety (ring A), most probably C-5 hydroxyl group is involved in the anodic reaction [7,25,26]. Besides above-mentioned, the oxidation of C-3 hydroxyl group (ring C) in **6** and **7** was found at less positive potential, *E*_p3_ ~ +0.37 V. The peaks occurred at less positive potentials indicate better antioxidant activity of attributed molecule part. Practically no difference in the potentials of oxidation between monomers and their respective dimers were observed (Table 1). This indicates that ability of the dimers to donate electron(s) is very similar as for the parent monomers. The reactivity and antioxidant capacity of the compounds investigated can be deduced from the potential of the first oxidation peak (wave) in the first anodic scan, where the antioxidant capacity of the oxidized compounds is negatively associated with their oxidation potential. Thus, the occurrence of peak 3 for **6** and **7** indicates that both compounds will be better antioxidants than **1a** and its dimers. This statement is in good agreement with many of biological studies where facilitation of biological activities and antioxidant capacity improvement was found for dehydroderivatives of flavonolignans in comparison to **1a** and its congeners [1]. In addition to redox properties, the adsorption behavior of **1a** and **6** was also studied and compared to the dimeric forms. It is well known that monomeric flavonolignans are strongly adsorbed onto electrodes at open circuit potential [7,27]. However, the adsorption of dimer **3** and **7** was strictly limited and practically impossible under the experimental conditions used (*cf.*Figure 2A,B). The differences in adsorption behavior were also confirmed by a.c. voltammetry (data not shown). Identical adsorption behavior can also be found for other dimers prepared in this work. All SWV data presented here were consistent with results acquired using cyclic voltammetry at *v* = 1 V s^−1^ with an *E*_p_ difference of ±0.05 V.

### 2.4. DPPH Scavenging

All the compounds except the intermediate **2a** were screened for their ability to scavenge a stable model radical, 1,1-diphenyl-2-picrylhydrazyl (DPPH) at 33 µM and they all were able to scavenge this radical with various efficiencies (Table 1). Silybin **1a **displayed 6.6% inhibition and values between 7.1% and 10.7% were found for its dimers, without significant difference (*p* < 0.05). In contrast, racemic 2,3-dehydrosilybin **6** scavenged 83% and its respected dimer **7** caused a 33.4% decrease in absorbance. Based on this screening and on the results from the voltammetry, we have selected representative dimers **3** and **7** and the parent molecules, **1a** and **6** for more detailed analyses. The IC_50_ for DPPH scavenging determined for **1a** was 225.4 ± 9.4 µM and a significant (*p* < 0.05) improvement in scavenging activity was noted for the respective dimer **3** with IC_50_ 137.1 ± 7.5 µM. The most powerful DPPH scavenger was racemic **6**, with IC_50_ 6.43 ± 0.30 µM. After dimerization, a significantly (*p* < 0.05) weaker activity was observed for the dimer **7**; IC_50_ 29.0 ± 0.7 µM.

### 2.5. Inhibition of Microsomal Lipid Peroxidation

**Figure 3:**
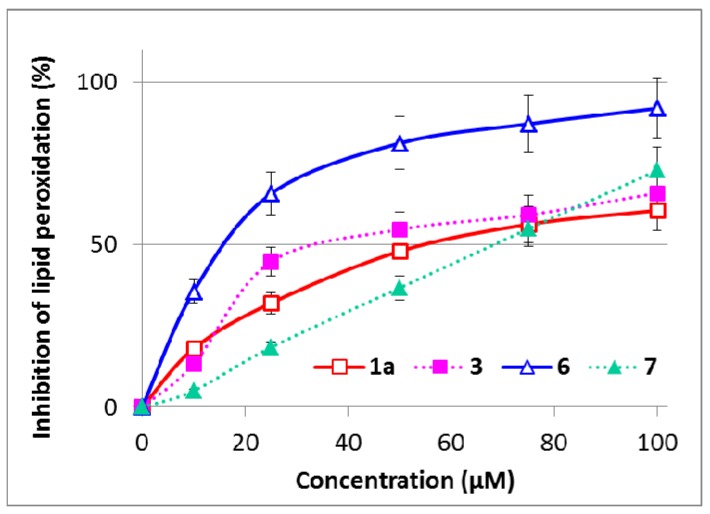
Effect of silybin and 2,3-dehydrosilybin dimers on inhibition of lipid peroxidation induced by 1 mM *tert*-butyl hydroperoxide in rat liver microsomes. The concentration of lipid peroxidation products was determined as thiobarbituric acid reactive substances (TBARS) and presented as %. Data are means ± SD from three independent experiments performed in triplicate.

All the compounds tested were able to inhibit the microsomal lipid peroxidation of rat liver microsomes, induced by *tert*-butyl hydroperoxide (Figure 3). The IC_50_ determined for **1a** was 53.7 ± 6.1 µM, while the antilipoperoxidant activity was improved (*p* < 0.05) for the respective dimer **3** with IC_50_ 34.0 ± 4.3 µM. The most powerful inhibitor of lipid peroxidation was again monomeric **6**, with IC_50_14.53 ± 0.73 µM. After dimerization, a weaker activity was observed for the dimer **7**; IC_50_ 59.2 ± 5.9 µM (*p* < 0.05). The lipid peroxidation data are in very good agreement with the DPPH test.

In both DPPH scavenging and microsomal lipid peroxidation tests, the data found for silybin A **1a** and 2,3-dehydrosilybin **6** are in good agreement with previously published data using a slightly different experimental setting [6,9,16,28,29]. With the dimers, we noted an activity for silybin dimer **3** that was approximately 1.6 times that of the parent molecule **1a**. This improvement could be attributed simply to an increased number of reactive OH-groups in the resulting molecule. In contrast, the dimerization of 2,3-dehydrosilybin resulted in about a quarter of the activity, which will be discussed in Section 2.6. The electrochemical results indicated that the redox behavior of monomeric and dimeric molecules is quite similar but the interaction (adsorption) ability of the dimers is different. So the explanation of the diversity in the DPPH scavenging and inhibition of lipid peroxidation of the tested flavonolignans will be probably based on their different intra- and inter-molecular interaction mode. This idea is supported by the fact that the oxidation mechanism (electron transfer ability) is practically the same for the monomeric and dimeric state of the compounds investigated.

To the best of our knowledge, no similar comparison has been performed to date. The silybin dimers prepared previously were not tested for biological activity [11,15]. No dimer of 2,3-dehydrosilybin has been prepared so far. Analogous dimers were prepared from ascorbic acid (and dehydroascorbic acid) but only the physico-chemical properties of the dimers were described [22]. Recently, a natural flavan-3-ol dimer was isolated from green tea from *Camellia taliensis* having DPPH- and ABTS^+^-scavenging activities superior to all other compounds from the tea, but unfortunately without testing the parent monomer [30]. Natural epicatechin dimer (procyanidin B2) exhibited, when the results were expressed based on a unit molar concentration of epicatechin, comparable DPPH and superoxide radical scavenging activities [31]. On the other hand, various dimers were identified as products of the reaction of various (poly)phenolics with DPPH [32,33,34].

**Figure 4:**
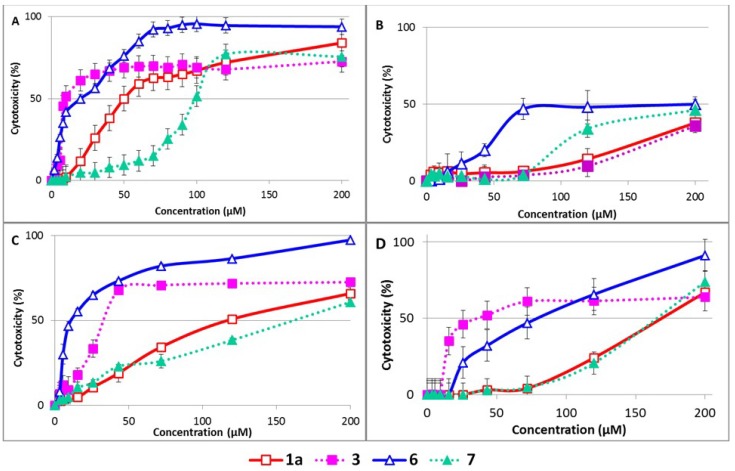
Effect of studied compounds on (**A**) human umbilical vein endothelial cells; (**B**) human normal adult keratinocytes; (**C**) mouse BALB/c fibroblasts and (**D**) HepG2 cells. The cells, grown to near confluence, were incubated with the tested compounds for 24 h and their viability was then assessed by MTT test. The results are presented as mean ± SD from three independent experiments performed in triplicate.

### 2.6. Cytotoxicity

The effect of the studied flavonolignans on cell cultures was evaluated in four types of cells. Two of them (HUVEC and NAK) are primary dividing cells with limited passage possibilities; HUVEC are vascular cells [35] and NAK are skin cells [36]. BALB/c 3T3 fibroblasts are an immortalized non-transformed cell line and HepG2 transformed hepatoma epithelial cells [37]. HUVEC were the most sensitive to the tested compounds and NAK were the most resistant, 50% cytotoxicity was not achieved for up to 200 µM of all the compounds tested in NAK cells (Figure 4). In all other cells used, the same trend can be observed. With **1a**, the parent compound displayed quite a low cytotoxicity (IC_50_ 60–166 µM) and its dimer **3** is much more cytotoxic (IC_50_ 8–44 µM). In contrast, 2,3-dehydrosilybin **6**, with an IC_50_ of 12–59 µM, is significantly more cytotoxic than the mixed 2,3-dehydrosilybin dimer (**7**), IC_50_ 99–173 µM, Table 2. These results follow a similar trend as the effect of the dimers on DPPH scavenging and microsomal lipoperoxidation. No data about the cytotoxicity of flavonolignan dimers are available in the literature. Nevertheless, a spontaneously formed heterodimer of the flavonol quercetin with quite a rigid structure was recently found to be responsible for its cytotoxicity towards MDA-MB-231 cells [38]. Biological activity towards P-glycoprotein-based multidrug resistance was evaluated for various synthetic luteolin dimers in multidrug resistant MDA435/LCC6 breast cancer cells and was found to be strongly influenced by the length of the linker used. Dimers with spacers between two and four ethylene glycols were effective at reversing multidrug resistance, while dimers with shorter or longer spacers exhibited little or no effect [21].

**Table 2:** Effect of selected compounds on viability of endothelial cells (HUVEC), keratinocytes (NAK), fibroblasts (BALB/c 3T3) and hepatoma epithelial cells (HepG2) ^a^.

	HUVEC	NAK	BALB/c 3T3	HepG2
**1a**	57.8 ± 3.6	>200	130.7 ± 8.0	165.4 ± 8.1
**3**	7.6 ± 1.9 ^b^	>200	23.5 ± 5.6 ^b^	51.8 ± 5.9 ^b^
**6**	20.9 ± 4.6	>200	11.9 ± 3.0	58.4 ± 9.0
**7**	98.4 ± 10.6 ^c^	>200	161.4 ± 9.0 ^c^	172.5 ± 11.6 ^c^

^a^ The cells were grown in 96-well plates to near confluence, then incubated with the tested compounds for 24 h and MTT-reducing ability was expressed as IC_50_ [µM] (mean ± SD from three independent experiments performed in triplicates); ^b^*p* < 0.05 compared with **1a**; ^c^*p* < 0.05 compared with **6**.

Taken together, these results led us to the hypothesis that in 2,3-dehydrosilybin **6**, dimerization using a flexible aliphatic ester linker probably resulted in the π-stacking of the two 2,3-dehydrosilybin units together and the blocking of some active groups. Similar inter and intra-molecular π-stacking was described, confirmed experimentally [39] and by means of molecular modelling [40,41] to be involved e.g., in copigmentation of anthocyanins and synthesis of oligostilbenoids [42]. Comparison of the hydration constants of various delphidin-based pigments revealed clearly that this type of interaction will be stronger for molecules with longer and more linear chains with greater flexibility. Moreover, a flat aromatic structure is required for π-stacking to occur [39]. Indeed, while the flavonoid part of 2,3-dehydrosilybin **6** was found to be planar, allowing π-electron delocalization, in silybin **1**, the planarity is lost with a strong distortion (*Φ* ca 74°) due to the loss of the 2,3-double bond and subsequently the loss of π-electron delocalization [16]. Π-stacking is, therefore, much more probable to occur in the dimer **7** compared to the compound **3**. In both tests, the DPPH and lipoperoxidation assays, the double bond between C-2 and C-3 and also unoccupied hydroxyl groups in positions C-3, C-5 and C-7 are essential for its high antioxidant properties. They play an important role in the mechanism of resonance stabilization which leads to a stable resonance structure [8,16]. The stacking of two units of 2,3-dehydrosilybin in the dimer molecule can block one or more hydroxyl groups and make resonance stabilization impossible, which probably decreases the reactivity and antioxidant activity of the 2,3-dehydrosilybin dimer.

## 3. Experimental

### 3.1. Chemicals and Reagents

Optically pure silybin A and B were prepared from commercial silymarin according to the published procedure [43]. *p*-Xylylene dibromide was obtained from ABCR (Karlsruhe, Germany). Other chemicals were obtained from Sigma-Aldrich (Prague, Czech Republic). Reactions were monitored by TLC (silica gel/TLC sheets 60 F_254_ Merck, Darmstadt, Germany). The lipase B from *Candida antarctica* immobilized on acrylic resin (Novozym 435) was purchased from Novo-Nordisk (Bagsvaerd, Denmark).

Dimethyl sulfoxide (DMSO) for cell cultures, 1,1-diphenyl-2-picrylhydrazyl (DPPH), Dulbecco’s modified Eagle’s medium (DMEM), MTT (3-(4,5-dimethyl-2-thiazolyl)-2,5-diphenyl-2*H*-tetrazolium bromide), streptomycin, penicillin, glutamine, trypsin-EDTA (0.25%), *tert*-butylhydroperoxide (*t*-BH, 70%), foetal calf serum (FCS) and newborn FCS were purchased from Sigma-Aldrich. Collagenase was from Serva (Heidelberg, Germany). Endothelial Cell Basal Medium with growth factors (Endothelial Growth Medium, EGM) was from Promocell (Heidelberg, Germany), EpiLife medium (M-EPI-500-CA) and Human Keratinocyte Growth Supplement (HKGS) from Life Technologies (Prague, Czech Republic). Other chemicals and solvents were of analytical grade from Pliva-Lachema (Brno, Czech Republic). For lipid peroxidation and cytotoxicity measurement, the tested compounds were dissolved in 10 mM of DMSO and these stock solutions were kept at −20 °C.

### 3.2. General Methods

NMR spectra were measured with a Bruker AVANCE III 600 MHz spectrometer (600.23 MHz for ^1^H, and 150.93 MHz for ^13^C) in DMSO-*d_6_*, 30 °C. The residual signal of the solvent was used as an internal standard (δ_H_ 3.330, δ_C_ 49.30). ^1^H-NMR, ^13^C-NMR, COSY, HSQC, and HMBC spectra were measured using the standard manufacturer’s software. Chemical shifts are given in δ-scale [ppm], and coupling constants in Hz. The digital resolution enabled us to report chemical shifts of protons to three and carbon chemical shifts to two decimal places. Some hydrogen chemical shifts were read out from the HSQC and are reported to two decimal places. Mass spectra were measured with a Micromass Platform LC system in methanol with the addition of formic acid. Reaction mixtures were incubated in a Thermomixer (Eppendorf, Hamburg, Germany).

### 3.3. Chemistry

#### 3.3.1. Synthesis of 12-Vinyl Dodecanedioate-23-*O*-Silybin B (**2b**)

Silybin B (**1b**, 1 mmol, 1 eq.) and divinylester of dodecanedioic acid [24] (2.624 mmol, 2.6 eq.) were dissolved in anhydrous acetonitrile (15 mL). Novozym 435 (200 mg) and 4 Å molecular sieves (200 mg) were added to the solution. The reaction mixture was incubated at 45 °C, 500 rpm. After 24 h, the reaction was terminated by filtering off the enzyme and the solvent was evaporated under reduced pressure. The crude product **2b** was purified by silica gel flash chromatography (chloroform/acetone 85:15).

*1-((3-(4-Hydroxy-3-methoxyphenyl)-6-(3,5,7-trihydroxy-4-oxochroman-2-yl)-2,3-dihydrobenzo[b]-[1,4]dioxin-2-yl)methyl) 12-vinyl dodecanedioate (12-vinyl dodecanedioate-23-*O*-silybin B)* (**2b**). White solid (yield 38%, 235 mg, 0.326 mmol). For ^1^H and ^13^C-NMR data, see Tables S1 and S2 in the Supporting Information. MS-ESI *m/z*: [M+H]^+^ Calcd. for C_39_H_45_O_13_ 721.5; Found: 721.3.

#### 3.3.2. General Procedure—Preparation of Diesters **3** and **4**

Silybin (**1a** or **1b**, 0.621 mmol, 2.7 eq.) and divinyldodecanedioate (0.230 mmol, 1 eq.) were dissolved in anhydrous acetonitrile (9 mL). Novozym 435 (150 mg) and 4 Å molecular sieves (50 mg) were added to the solution. The reaction mixture was incubated at 45 °C, 500 rpm. After 72 h, the reaction was stopped by filtering off the enzyme and the solvent was evaporated under reduced pressure. The crude product was purified by gel filtration using Sephadex LH 20 column, 140 × 1.8 cm (eluent MeOH/H_2_O 4:1).

*bis(((2R,3R)-3-(4-Hydroxy-3-methoxyphenyl)-6-((2R,3R)-3,5,7-trihydroxy-4-oxochroman-2-yl)-2,3-dihydrobenzo[b][1,4]dioxin-2-yl)methyl) dodecanedioate* (**3**). White solid (yield 24%, 86 mg). For ^1^H and ^13^C-NMR data, see Tables S1 and S2 in the Supporting Information. MS-ESI *m/z*: [M+H]^+^ Calcd. for C_62_H_63_O_22_ 1158.4; Found 1158.3.

*bis(((2S,3S)-3-(4-Hydroxy-3-methoxyphenyl)-6-((2R,3R)-3,5,7-trihydroxy-4-oxochroman-2-yl)-2,3-dihydrobenzo[b][1,4]dioxin-2-yl)methyl) dodecanedioate* (**4**). White solid (yield 44%, 118 mg). For ^1^H and ^13^C-NMR data, see Tables S1 and S2 in the Supporting Information. MS-ESI *m/z*: [M+H]^+^ Calcd. for C_62_H_63_O_22_ 1158.4; Found 1158.4.

#### 3.3.3. Preparation of Mixed Dimer **5**

12-Vinyl dodecanedioate-23-*O*-silybin B (**2b**, 0.278 mmol, 1 eq.) and silybin A (**1a**, 0.556 mmol, 2 eq.) were dissolved in anhydrous acetonitrile (9 mL). Novozym 435 (100 mg) and 4 Å molecular sieves (100 mg) were added to the solution. The reaction mixture was incubated at 45 °C, 500 rpm. After 72 h, the reaction was stopped by filtering off the enzyme and the solvent was evaporated under reduced pressure. The crude product was purified by gel filtration using Sephadex LH 20 column 140 × 1.8 cm (eluent MeOH/H_2_O 4:1).

*1-(((2R,3R)-3-(4-Hydroxy-3-methoxyphenyl)-6-((2R,3R)-3,5,7-trihydroxy-4-oxochroman-2-yl)-2,3-dihydrobenzo[b][1,4]dioxin-2-yl)methyl) 12-(((2S,3S)-3-(4-hydroxy-3-methoxyphenyl)-6-((2R,3R)-3,5,7-trihydroxy-4-oxochroman-2-yl)-2,3-dihydrobenzo[b][1,4]dioxin-2-yl)methyl) dodecanedioate* (**5**). White solid (yield 26%, 85 mg). For ^1^H and ^13^C-NMR data, see Tables S1 and S2 in the Supporting Information. MS-ESI *m/z*: [M+H]^+^ Calcd. for C_62_H_63_O_22_ 1158.1; Found 1158.1.

#### 3.3.4. Preparation 2,3-Dehydrosilybin Dimer **7**

2,3-Dehydrosilybin (**6**, 0.416 mmol, 4 eq.) and the divinylester of dodecanedioic acid (0.104 mmol, 1 eq.) were dissolved in anhydrous acetone (20 mL). Novozym 435 (200 mg) and 4 Å molecular sieves (50 mg) were added to the solution. The reaction mixture was incubated at 45 °C, 500. After 16 h, the reaction was stopped by filtering off the enzyme and the solvent was evaporated under reduced pressure. The crude product was purified by flash chromatography (CHCl_3_/acetone 4:1).

*bis((3-(4-Hydroxy-3-methoxyphenyl)-6-(3,5,7-trihydroxy-4-oxo-4H-chromen-2-yl)-2,3-dihydrobenzo-[b][1,4]dioxin-2-yl)methyl) dodecanedioate* (**7**). Yellow solid (yield 19%, 90 mg). For ^1^H and ^13^C-NMR data, see the Supporting Information. MS-ESI *m/z*: [M+H]^+^ Calcd. for C_62_H_58_O_22_ 1153.8; Found 1153.8. HRMS [M+Na]^+^ Calcd. 1177.3317; Found 1177.3328.

#### 3.3.5. General Procedure: Preparation of Silybin Diethers **8**, **9**, **10** and **11**

K_2_CO_3_ (5.8 mmol, 7 eq.) and *p*-xylylene dibromide (0.42 mmol, 0.5 eq.) or *m*-xylylene dibromide (0.42 mmol, 0.5 eq.) were added to the solution of silybin (**1** or **1a** or **1b**, 400 mg, 0.83 mmol, 1 eq.) in dry acetone (20 mL). The mixture was stirred and refluxed for 4 h under nitrogen. The reaction was quenched by the addition of conc. HCl (1 mL), diluted with water (50 mL) and extracted with ethyl acetate (2 × 30 mL). The organic layer was dried over Na_2_SO_4_ and evaporated. The residue was purified by flash chromatography (linear gradient from chloroform/acetone 85:15 to acetone 100%) and the title compound was isolated as a white amorphous solid.

*(2R,2'R,3R,3'R)-7,7'-((1,4-Phenylenebis(methylene))bis(oxy))bis(3,5-dihydroxy-2-((2R,3R)-3-(4-hydroxy-3-methoxyphenyl)-2-(hydroxymethyl)-2,3-dihydrobenzo[b][1,4]dioxin-6-yl)chroman-4-one)* (**8**). This compound was prepared according to the general procedure from **1a** and *p*-xylylene dibromide. Product was isolated as a white solid (yield 7%, 30 mg). For ^1^H and ^13^C-NMR data, see Tables S1 and S3 in the Supporting Information. MS-ESI *m/z*: [M−H]^+^ Calcd. for C_58_H_50_O_20_1065.5; Found 1065.5.

*(2R,2'R,3R,3'R)-7,7'-((1,4-Phenylenebis(methylene))bis(oxy))bis(3,5-dihydroxy-2-((2S,3S)-3-(4-hydroxy-3-methoxyphenyl)-2-(hydroxymethyl)-2,3-dihydrobenzo[b][1,4]dioxin-6-yl)chroman-4-one)* (**9**). This compound was prepared according to the general procedure from **1b** and *p*-xylylene dibromide. Product was isolated as a white solid (yield 9%, 35 mg). For ^1^H and ^13^C-NMR data, see Tables S1 and S3 in the Supporting Information. MS-ESI *m/z*: [M−H]^+^ Calcd. for C_58_H_50_O_20_ 1065.4; Found 1065.4.

*(2R,2'R,3R,3'R)-7,7'-((1,4-Phenylenebis(methylene))bis(oxy))bis(3,5-dihydroxy-2-(3-(4-hydroxy-3-methoxyphenyl)-2-(hydroxymethyl)-2,3-dihydrobenzo[b][1,4]dioxin-6-yl)chroman-4-one)* (**10**). This compound was prepared according to the general procedure from **1** and *p*-xylylene dibromide. Product was isolated as a white solid (yield 15%, 40 mg). For ^1^H and ^13^C-NMR data, see Tables S1 and S3 in the Supporting Information. MS-ESI *m/z*: [M−H]^+^ Calcd. for C_58_H_50_O_20_ 1065.5; Found 1065.3.

*(2R,2'R,3R,3'R)-7,7'-((1,3-Phenylenebis(methylene))bis(oxy))bis(3,5-dihydroxy-2-(3-(4-hydroxy-3-methoxyphenyl)-2-(hydroxymethyl)-2,3-dihydrobenzo[b][1,4]dioxin-6-yl)chroman-4-one)* (**11**). This compound was prepared according to the general procedure from **1** and *m*-xylylene dibromide. Product was isolated as a white solid (yield 14%, 30 mg). For ^1^H and ^13^C-NMR data, see Tables S1 and S3 in the Supporting Information. MS-ESI *m/z*: [M−H]^+^ Calcd. for C_58_H_50_O_20_ 1065.5; Found 1065.5.

### 3.4. Stability of Dimers—HPLC Analysis

The stability of the dimers in PBS buffer and phosphate buffer (0.2 M, pH 7.4) was monitored using an HPLC method. 40 mM solutions of compounds **4** and **10** in DMSO were diluted with 2 mL of each buffers and incubated for 24 h at room temperature. The chromatography was carried out in a Shimadzu Prominence UFLC system (Kyoto, Japan) consisting of a DGU-20A mobile phase degasser, two LC-20AD solvent delivery units, SIL-20ACHT cooling autosampler, CTO-10AS column oven and SPD-M20A (PDA, photodiode array detector). A Chromolith Performance RP-18e monolithic column (100 × 3 mm i.d.) and guard column (5 × 4.6 mm i.d., both Merck) were used. The PDA data were acquired in the 200–450 nm range and the 285 signal was extracted. Gradient elution: mobile phase A: 0.1% formic acid, 10% methanol in water (v/v/v); mobile phase B: 0.1% formic acid in methanol (*v/v*); gradient, 0–1 min, 20% B; 1–8 min, 20%–90% B; 8–9 min, 90% B, 9–10 min, 90%–20% B. The flow rate was 1.2 mL/min at 25 °C.

### 3.5. Electrochemical Measurement

The monomeric flavonolignans and their dimers were analyzed using *in situ* voltammetry with the working electrode (PGE: pyrolytic graphite electrode) dipped in the supporting electrolyte containing the analytes and/or *ex situ* voltammetric analysis (adsorptive transfer, AdT technique). For this purpose, the PGE was first dipped into a 5 µL aliquot of the studied sample. After an accumulation period (30 s), the electrode was washed with deionized water and placed in an electrochemical cell containing pure supporting electrolyte. Square-wave voltammetry (SWV) and cyclic voltammetry (CV) were performed at room temperature with a µAutolab III analyzer (EcoChemie, Utrecht, The Netherlands) in a three-electrode setup (Ag/AgCl 3M KCl electrode as a reference and a platinum wire as the auxiliary electrode). SWV parameters: frequency: 200 Hz, from 0 V to +1.5 V, concentration of tested compounds (50 µM), supporting electrolyte: Britton-Robinson buffer, pH 7.4. The flavonolignans analyzed were dissolved in methanol as a stock solution of 1 mg mL^−1^ (stored in a fridge in the dark), and diluted with the supporting electrolyte and/or 0.2 M acetate buffer (pH 5) for *in situ* and/or *ex situ* voltammetry. *Ex situ* a.c. voltammetry (out-of-phase) was carried out with a hanging mercury drop electrode (HMDE) according to the previously published paper [44]. In this case, the flavonolignans (50 µM) were adsorbed from 0.2 M acetate buffer (pH 5) for 30 s. For current responses and potentials (*E*_p_), the standard deviations (S.D.) were less than ±3.55% and ±3 mV for all experiments, respectively.

### 3.6. DPPH Assay

Antiradical activity was evaluated spectrophotometrically as the ability of the tested substances to reduce the 1,1-diphenyl-2-picrylhydrazyl (DPPH) radical as described previously [45] with minor modifications. Briefly, a solution of the tested substance (10 µL, final concentration 0–540 µM) were mixed with a freshly prepared methanolic DPPH solution (290 µL, final concentration 20 µM) in a microtiter plate well. After 30 min, absorbance at 517 nm was measured, percentages of inhibition were calculated using the negative control and for selected compounds the IC_50_ values were obtained from the inhibition curves.

### 3.7. Inhibition of Microsomal Lipid Peroxidation

Microsomes were prepared from rat liver homogenate as described previously [46] and resuspended in 50 mM Tris-HCl buffer with 100 mM KCl and 0.1 mM EDTA (pH 7.4). The protein concentration in the stock microsomal suspension was determined using the Bradford method [47] to be typically ca 30 mg protein/mL. This stock suspension was stored at −80 °C and diluted to 0.625 mg protein/mL using PBS before use. 0.4 mL of the diluted microsomal suspension was then mixed with the compounds tested (final concentration 10–200 µM in 0.01 mL DMSO) and incubated for 10 min at 37 °C. *tert*-Butyl hydroperoxide (0.09 mL in PBS; final concentration 1 mM) was then added and the mixture was incubated at 37 °C for 30 min. The products of lipid peroxidation were determined as thiobarbituric acid reactive substances (TBARS): 0.7 mL of trichloroacetic acid (26 mM) with thiobarbituric acid (918 mM) were added, the mixture was heated (90 °C; 30 min), cooled, centrifuged (10 min; 10,000 rpm; 4 °C) and the absorbance of the supernatant at 535 nm measured. The activity was calculated as the concentration of the tested compound that inhibited the color reaction with thiobarbiturate (without the tested compound) by 50% (IC_50_).

### 3.8. Source of Human Tissues

Human umbilical cords and skin samples were obtained from healthy volunteers, who had to fulfill the requirements for submission to the study and sign a personal awareness declaration. The choice of volunteers was in accordance with the principles of the International Ethics Committee for Biomedical Research [48] and the study was approved by the Ethics Commission of Palacký University and University Hospital in Olomouc.

### 3.9. Cell Cultures

All the cells were cultured in a humidified atmosphere with 5% (*v/v*) CO_2_ at 37 °C and subcultured before conﬂuence. Human Umbilical Vein Endothelial Cells (HUVEC) were isolated from umbilical cords of healthy non-smoker women (18–35 years) as described previously [49], cultivated in gelatin-coated flasks in EGM and used in passages 2–6. Primary Normal human Adult epidermal Keratinocytes (NAK) were isolated from normal skin obtained from excessive tissue removed during abdominoplasty or mammoplasty of healthy adult patients as described in [36,50], cultivated in EpiLife medium with the addition of HKGS. Mouse fibroblasts (BALB/c 3T3 cells) were purchased from the European Collection of Cell Cultures (ECACC, Salisbury, UK) and were grown in DMEM supplemented with heat-inactivated FCS (5%, *v/v*) and newborn FCS, streptomycin (100 U/mL), penicillin (0.1 mg/mL) and glutamine (4 mM). Hepatocyte carcinoma HepG2 cells (No. 85011430, ECACC) were cultured at 37 °C in DMEM supplemented with 2 mM L-glutamine, 1% non-essential amino acids, 100 U/mL penicillin, 100 mg/mL streptomycin and 10% fetal bovine serum (Invitrogen, Carlsbad, CA, USA).

### 3.10. Cytotoxicity Testing

The cells were plated into 96-well cell culture plates and grown to near confluence. The tested compounds were placed in the respective fresh medium in serial dilutions (100 µL/well) and incubated with the cells for 24 h. After incubation, the medium was removed, the cells were washed with PBS, fresh medium containing 1 mg/mL of MTT was added and the plates were incubated for a further 3 h (37 °C). The resulting formazan was dissolved in 50 µL of 1% NH_3_ in DMSO, absorbance was read at 540 nm and IC_50_ values were obtained graphically from the dependence of cell viability on the concentration of the tested compound.

### 3.11. Statistical Analysis

Data were analyzed with one-way ANOVA using the statistical package statext ver. 2.1. Differences were considered statistically significant when *p* < 0.05.

## 4. Conclusions

Divalent or multivalent molecules can modulate and promote biological activity compared to their monomers. Two different types of silybin or 2,3-dehydrosilybin dimers, differing in the connection of their monomeric units, were prepared and characterized. Electrochemical measurements show that mechanism of oxidation of all dimeric compounds (*vs*. parent molecules) is probably the same. The specific adsorption behavior onto the electrode surface indicates that the dimers could probably specifically interact with biomacromolecules and membranes and that this would influence their reactivity and uptake into cells, which was confirmed by various biological *in vitro* tests. The DPPH-scavenging and inhibition of microsomal lipid peroxidation was slightly improved by dimerization with silybin; but the dimer exhibited a higher cytotoxicity (HUVEC, BALB/c 3T3, HepG2) than the parent compound. In contrast to this, the dimer of 2,3-dehydrosilybin exhibited a significant lowering of both DPPH-scavenging and antilipoperoxidant activity, and a lower cytotoxicity compared to 2,3-dehydrosilybin. This behavior might be caused by the stacking of the aromatic 2,3-dehydrosilybin moieties in the dimer. We assume that knowledge on the basic redox, antioxidant and toxicological properties of the novel flavonolignan dimers presented here can be applied in subsequent more complex biological studies.

## Supplementary Materials

Supplementary data containing ^1^H and ^13^C-NMR and MS data associated with this article can be found, in the online version, at: http://www.mdpi.com/1420-3049/19/4/4115/s1.
