# Supplementary material for: Chemo-Enzymatic Synthesis of Silybin and 2,3-Dehydrosilybin Dimers
Source: Molecules. 2014 Apr 2;19(4):4115–34. doi: 10.3390/molecules19044115 (PMC6271273; doi:10.3390/molecules19044115)
Supplement: Supplementary file 1 [file molecules-19-04115-s001.pdf]

Supplementary Materials

Table S1. ^{13}C-NMR data of prepared compounds ($\delta_{C,}$ ppm).

Atom #	Compound							
	**2b** [1]	**3** [2]	**4** [2]	**5** [2]	**8** [1]	**9** [1]	**10** [3]	**11** [1]
**2**	82.48	82.49	82.45	82.52	82.74	82.72	82.74	82.77
	82.53			82.47			82.71	82.73
**3**	71.55	71.43	71.50	71.52	71.50	71.58	71.57	71.60
	71.47			71.44			71.50	71.52
**4**	197.73	197.69	197.62	197.78	198.44	198.41	198.40	198.44
	197.76			197.75			198.38	198.41
**4a**	100.51	100.48	100.45	100.52	101.55	101.56	101.55	101.58
**5**	163.36	163.31	163.33	163.35	162.97	162.99	162.98	163.00
**6**	96.15	96.10	96.12	96.12	95.77	95.78	95.77	95.78
**7**	166.93	166.84	166.95	166.86	166.50	166.52	166.50	166.54
**8**	95.10	95.05	95.08	95.08	94.62	94.63	94.62	94.59
**8a**	162.49	162.47	162.45	162.50	162.44	162.43	162.43	162.46
	162.50			162.49			162.41	162.44
**10**	75.06	75.00	75.02	75.04	78.15	78.18	78.17	78.19
	75.04			75.02			78.16	78.16
**11**	75.98	75.94	75.95	75.97	75.88	75.90	75.88	75.90
**12a**	143.24	143.22	143.20	143.26	143.31	143.30	143.30	143.32
	143.27			143.24			143.28	143.30
**13**	116.83	116.67	116.77	116.83	116.65	116.72	116.70	116.72
	116.71			116.72			116.63	116.66
**14**	130.59	130.50	130.55	130.58	129.98	129.95	129.93	129.90
	130.55			130.54			129.89	129.85
**15**	121.38	121.53	121.35	121.60	121.41	121.26	121.39	121.42
	121.59			121.41			121.24	121.26
**16**	116.46	116.37	116.42	116.46	116.36	116.42	116.40	116.43
	116.40			116.41			116.36	116.38
**16a**	143.11	143.10	143.06	143.13	143.75	143.73	143.74	143.76
	143.14			143.10			143.71	143.73
**17**	126.64	126.61	126.62	126.64	127.53	127.55	127.54	127.55
	126.63							
**18**	111.71	111.78	111.74	111.75	111.78	111.73	111.80	111.79
	111.76			111.70			111.75	111.73
**19**	147.84	147.79	147.80	147. 82	147.67	147.70	147.68	147.70
	147.83			147.81			147.67	147.69
**20**	147.40	147.36	147.36	147.37	147.07	147.07	147.07	147.08
	147.41							147.69
**21**	115.45	115.44	115.43	115.44	115.37	115.37	115.37	115.39
	115.46							115.37
**22**	120.64	120.64	120.62	120.65	120.57	120.57	120.55	120.57
	120.67							
**23**	62.45	62.42	62.42	62.46	60.23	60.23	60.22	60.24
**19-MeO**	55.73	55.73	55.73	55.73	55.75	55.76	55.76	55.76
**1'**	172.63	172.58	172.59	172.64	69.55	69.56	69.55	69.74
**2'**	32.26	33.22	33.22	33.24	136.13	136.14	136.12	136.63
**3'**	24.41	24.36	24.36	24.40	127.98	127.99	127.96	127.06
**4'**	28.46	28.42	28.43	28.47				127.61

Table S1. *Cont.*

**Atom #**	**Compound**							
	**2b** [1]	**3** [2]	**4** [2]	**5** [2]	**8** [1]	**9** [1]	**10** [3]	**11** [1]
**5'**	28.69	28.66	28.66	28.71				128.86
**6'**	28.85	28.85	28.85	28.90				
**7'**	28.83							
**8'**	28.65							
**9'**	28.38							
**10'**	24.10							
**11'**	33.09							
**12'**	170.48							
**1''**	141.30							
**2''**	98.01							

[1] Bruker Avance III 700 (700.13 MHz for ^{1}H, 176.05 MHz for ^{13}C, DMSO-d_6, 303.2 K); [2] Bruker Avance III 600 (600.23 MHz for ^{1}H, 150.94 MHz for ^{13}C, DMSO-d_6, 303.2 K); [3] Bruker Avance III 400 (400.00 MHz for ^{1}H, 100.58 MHz for ^{13}C, DMSO-d_6, 303.2 K).

Table S2. ^{1}H-NMR data of prepared compounds.

**Atom #**	**2b** [1]			**3** [3]			**4** [2]			**5** [2]		
	**δ_H [ppm]**	**m** [#]	**$J_{H\text{-}H}$ [Hz]**	**δ_H [ppm]**	**m** [#]	**$J_{H\text{-}H}$ [Hz]**	**δ_H [ppm]**	**m**[#]	**$J_{H\text{-}H}$ [Hz]**	**δ_H [ppm]**	**m** [#]	**$J_{H\text{-}H}$ [Hz]**
**2**	5.091	d	11.2	5.088	d	11.2	5.086	d	11.2	5.088	d	11.2
**3**	4.602	dd	5.9, 11.2	4.597	dd	6.2, 11.2	4.582	dd	6.0, 11.2	4.598	m	-
	4.588	dd	5.9, 11.2									
**6**	5.924	d	2.0	5.913	d	2.1	5.910	d	2.1	5.912	m	-
**8**	5.881	d	2.0	5.866	d	2.1	5.868	d	2.1	5.868	m	-
	5.874	d	2.0									
**10**	4.514	m	-	4.506	ddd	2.8, 5.0, 7.9	4.496	ddd	2.8, 5.0, 7.9	4.504	m	-
	4.503	m	-									
**11**	4.928	d	7.9	4.920	d	7.9	4.922	d	7.9	4.922	d	8.0
**13**	7.106	d	1.9	7.108	d	2.0	7.100	d	2.0	7.112	d	2.0
	7.117	d	1.9							7.102	d	2.0
**15**	7.034[J]	dd	1.9, 8.3	7.023	dd	2.0, 8.3	7.029	dd	2.0, 8.3	7.029	dd	2.0, 8.3
	7.027[J]	dd	1.9, 8.2							7.022	dd	2.0, 8.3
**16**	6.984	d	8.3	6.975	d	8.3	6.979	d	8.3	6.981	d	8.3
	6.979	d	8.2							6.977	d	8.3
**18**	7.023[J]	d	1.8	7.014	d	1.9	7.017	d	2.0	7.01 *	m	-
	7.020[J]	d	1.8									
**21**	6.806	d	8.1	6.799	d	8.1	6.800	d	8.0	6.854	dd	1.9, 8.1
**22**	6.860	dd	1.8, 8.1	6.855	dd	1.9, 8.1	6.855	dd	2.0, 8.0	6.797	d	8.1
										6.796	d	8.1
**23**	4.133	dd	2.5, 12.4	4.126	dd	2.8, 12.4	4.126	dd	2.8, 12.4	4.928	dd	5.0, 12.5
	3.934	dd	4.9, 12.4	3.934	dd	5.0, 12.4	3.933	dd	5.0, 12.4	4.120	dd	2.6, 12.5
**3-OH**	5.091	d	5.9	5.774	d	6.2	5.770	d	6.0	5.794	d	6.2

Table S2. *Cont.*

**Atom #**	**2b** [1]			**3** [3]			**4** [2]			**5** [2]		
	**δ_H [ppm]**	**m** [#]	**J_{H-H} [Hz]**	**δ_H [ppm]**	**m** [#]	**J_{H-H} [Hz]**	**δ_H [ppm]**	**m**[#]	**J_{H-H} [Hz]**	**δ_H [ppm]**	**m** [#]	**J_{H-H} [Hz]**
**5-OH**	11.876	s	-	11.859	s	-	11.863	s	-	11.871	s	-
**7-OH**	10.861	s	-	10.794	s	-	10.813	s	-	10.818	s	-
										10.814	s	-
**19-OMe**	3.777	s	-	3.769	s	-	3.772	s	-	3.770	s	-
	3.774	s	-							3.767	s	-
**20-OH**	9.192	s	-	9.157	s	-	9.157	s	-	9.185	s	-
										9.184	s	-
**2'**	2.318	dt	16.0, 7.5	2.293	m	-	2.294	m	-	2.296	m	-
	2.284	dt	16.0, 7.5									
**3'**	1.49^{H}	m	-	1.490	m	-	1.491	m	-	1.488	m	-
**4'**	1.24^{H}	m	-									
**5'**				1.235	m	-	1.235	m	-	1.23^{H}	m	-
**6'**	1.23^{H}–1.25^{H}	m	-									
**7'**												-
**8'**												
**9'**	1.25^{H}	m	-									
**10'**	1.54^{H}	m	-									
**11''**	2.403	t	7.3									
**1''**	7.207	dd	6.2, 14.0									
**2''**	4.875	dd	14.0, 1.5									
	4.630	dd	6.2, 1.5									

J—J-RES readout; H—HSQC readout; [1] Bruker Avance III 700 (700.13 MHz for ^{1}H, 176.05 MHz for ^{13}C, DMSO-d_6, 303.2 K); [2] Bruker Avance III 600 (600.23 MHz for ^{1}H, 150.94 MHz for ^{13}C, DMSO-d_6, 303.2 K); [3] Bruker Avance III 400 (400.00 MHz for ^{1}H, 100.58 MHz for ^{13}C, DMSO-d_6, 303.2 K).

Table S3. ^{1}H-NMR data of prepared compounds.

**Atom #**	**8** [1]			**9** [1]			**10** [3]			**11** [1]		
	**δ_H [ppm]**	**m** [#]	**J_{H-H} [Hz]**	**δ_H [ppm]**	**m** [#]	**J_{H-H} [Hz]**	**δ_H [ppm]**	**m** [#]	**J_{H-H} [Hz]**	**δ_H [ppm]**	**m** [#]	**J_{H-H} [Hz]**
**2**	5.131	d	11.3	5.132	d	11.3	5.132	d	11.3	5.134	d	11.3
**3**	4.669	dd	6.3, 11.3	4.659	dd	6.3, 11.3	4.668	dd	6.3, 11.3	4.666	m	-
							4.655	dd	6.3, 11.3			
**6**	6.184	d	2.2	6.118	d	2.2	6.187	d	2.0	6.19[H]	m	-
**8**	6.157	d	2.2	6.164	d	2.2	6.164	d	2.0	6.17[H]	m	-
							6.157	d	2.0			
**10**	4.172	ddd	2.5, 4.5, 7.9	4.166	ddd	2.5, 4.7, 7.8	4.173	m	-	4.17[H]	m	-
							4.165	m				
**11**	4.910	d	7.9	4.913	d	7.8	4.913	d	8.0	4.911	d	7.8
**13**	7.089	d	2.0	7.086	d	2.0	7.090	d	2.0	7.093	d	1.9
							7.084	d	2.0	7.087		
**15**	7.013	dd	2.0, 8.3	7.019	dd	2.0, 8.3	7.020	m	-	7.02[H]	m	-
							7.015	m				
**16**	6.970	d	8.3	6.975	d	8.3	6.972	d	8.4	6.974	d	8.3
							6.969	d	8.2	6.972	d	
**18**	7.010	d	1.9	7.016	d	1.8	7.015	d	1.9	7.01[H]	m	-
							7.011	d	1.9			
**21**	6.802	d	8.2	6.804	d	8.1	6.802	d	8.1	6.804	d	8.0
										6.803	d	

Table S3. *Cont.*

Atom #	**8** [1]			**9** [1]			**10** [3]			**11** [1]		
	**δ_H [ppm]**	**m** [#]	**J_{H-H} [Hz]**	**δ_H [ppm]**	**m** [#]	**J_{H-H} [Hz]**	**δ_H [ppm]**	**m** [#]	**J_{H-H} [Hz]**	**δ_H [ppm]**	**m** [#]	**J_{H-H} [Hz]**
**22**	6.861	d	1.9, 8.2	6.865	dd	1.8, 8.1	6.865	dd	8.1, 1.9	6.864	m	-
**23**	3.541	ddd	2.5, 4.9, 12.3	3.543	ddd	2.5, 5.0, 12.3	3.544	ddd	2.6, 5.0, 12.2	3.54[H]	m	-
	3.347	ddd	4.5, 5.8, 12.3	3.350	ddd	4.7, 5.7, 12.3	3.351	ddd	4.7, 5.8, 12.2	3.35[H]	m	-
**3-OH**	5.849	d	6.3	5.854	d	6.3	5.842	d	6.3	5.857	d	6.3
**5-OH**	11.819	s	-	11.824	s	-	11.818	s	-	11.826	s	-
**19-OMe**	3.775	s	-	3.778	s	-	3.778	s	-	3.776	s	-
**20-OH**	9.120	s	-	9.122	s	-	9.108	s	-	9.122	s	-
							9.106	s	-			
**23-OH**	4.931	dd	4.9, 5.8	4.938	dd	5.0, 5.7	4.923	dd	5.0, 5.8	4.936	m	
**1'**	5.171	s	-	5.176	s	-	5.175	s	-	5.178	s	-
**3'**	7.433	s	-	7.437	s	-	7.444	s	-	7.48[H]	m	-
							7.435	s				
**4'**										7.400	m	-
**5'**				5.854	d	6.3				7.410	m	-

J—J-RES readout; H—HSQC readout; [1] Bruker Avance III 700 (700.13 MHz for ^{1}H, 176.05 MHz for ^{13}C, DMSO-d_6, 303.2 K); [2] Bruker Avance III 600 (600.23 MHz for ^{1}H, 150.94 MHz for ^{13}C, DMSO-d_6, 303.2 K); [3] Bruker Avance III 400 (400.00 MHz for ^{1}H, 100.58 MHz for ^{13}C, DMSO-d_6, 303.2 K).

^{1}H-NMR data of prepared compound 7

^{1}H-NMR (600.13 MHz, CD_3OD, 20 °C) δ: 1.31[T] (m, H-4’, H-9’), 1.62[T] (m, H-3’, H-10'), 2.356 (m, H-2', H-11'), 3.910 (s, 19-OMe), 3.981 (m, H-23u), 4.405 (m, H-23d), 4.448 (m, H-10), 4.96[H] (m, H-11), 6.200 (br s, H-6), 6.439 (br s, H-8), 6.887 (d, *J* = 8.1 Hz, H-21), 6.943 (dd, *J* = 1.9, 8.1 Hz, H-22), 7.055 (d, *J* = 1.9 Hz, H-18), 7.080 (m, H-16), 7.842 (m, H-15), 7.898 (m, H-13).

[T] TOCSY readout
